# The Role of Vitamins in Spinal Cord Injury: Mechanisms and Benefits

**DOI:** 10.1155/2024/4293391

**Published:** 2024-06-20

**Authors:** Fatemeh Abbaszadeh, Pegah Javadpour, Mohammad Mehdi Mousavi Nasab, Masoumeh Jorjani

**Affiliations:** ^1^ Neurobiology Research Center Shahid Beheshti University of Medical Sciences, Tehran, Iran; ^2^ Neuroscience Research Center Shahid Beheshti University of Medical Sciences, Tehran, Iran; ^3^ School of Medicine Shahid Beheshti University of Medical Sciences, Tehran, Iran; ^4^ Department of Pharmacology School of Medicine Shahid Beheshti University of Medical Sciences, Tehran, Iran

## Abstract

Spinal cord injury (SCI) is a common neurological disease worldwide, often resulting in a substantial decrease in quality of life, disability, and in severe cases, even death. Unfortunately, there is currently no effective treatment for this disease. Nevertheless, current basic and clinical evidence suggests that vitamins, with their antioxidant properties and biological functions, may play a valuable role in improving the quality of life for individuals with SCI. They can promote overall health and facilitate the healing process. In this review, we discuss the mechanisms and therapeutic potential of vitamins in the treatment of SCI.

## 1. Introduction

Spinal cord injury (SCI) can result from trauma or spinal cord disease, leading to a wide range of neurological disorders and, in severe cases, even death. The pathophysiological mechanisms of SCI include primary and secondary injury processes. The primary mechanical injury, involving the physical disruption of neural tissue, sets off a chain of biochemical and metabolic events that ultimately lead to secondary injury and cell death. Secondary damage includes a cascade of critical events such as the formation of glial scar, inflammation, edema, ischemia, oxidative stress, and apoptosis. These pathophysiological mechanisms are interconnected and can exacerbate one another, resulting in further damage and functional impairment in individuals with SCI [1].

The extent and level of the injury can determine the range of morbidities associated with SCI. These comorbidities may include motor paralysis, sensory loss, pain, osteoporosis, end organ dysfunction, obesity, diabetes, sexual dysfunction, immune system disorders, and more. These comorbidities can significantly impact a person's quality of life and require specialized care and management [2, 3, 4].

Various treatments are available to manage symptoms and enhance the overall quality of for individuals living with SCI. One promising treatment approach involves the use of vitamins with antioxidant, anti-inflammatory, and neuroprotective properties [5, 6]. Vitamins are naturally occurring substances that play a crucial role in maintaining optimal human body functions. Since the body cannot synthesize these essential nutrients, they must be obtained from the diet. A deficiency in vitamins can exacerbate SCI symptoms. Vitamins are classified into two groups: fat-soluble vitamins (A, D, E, and K), which primarily interact with cellular nuclear receptors, influencing the expression of specific genes, and water-soluble vitamins (B and C), which mainly function as enzyme cofactors [7, 8]. Vitamin levels in people with SCI may be linked to overall health, functional abilities, and the risk of developing pressure ulcers [9]. Vitamins can aid in managing SCI through several mechanisms. In this review, we summarize the underlying pathophysiological mechanisms of SCI and discuss the therapeutic potential of vitamins in its treatment.

## 2. Oxidative Stress after SCI

Oxidative stress is a pathological condition characterized by an imbalance between the production of reactive oxygen species (ROS) and the body's ability to counteract them through antioxidant defense mechanisms. In SCI, several mechanisms contribute to oxidative stress, including the release of proinflammatory cytokines, activation of microglia and astrocytes, and mitochondrial dysfunction. Excessive ROS further exacerbate excitotoxicity and mitochondrial dysfunction, ultimately leading to neuronal cell death. These mechanisms results in the generation of both ROS and reactive nitrogen species (RNS), triggering a cascade of degenerative processes that impair ATP production and harm spinal cord tissue, intensifying the primary injury [10]. Oxidative stress and inflammation are closely interrelated processes in SCI. ROS can activate nuclear factor-kappa B (NF-*κ*B), a transcription factor that regulates the expression of proinflammatory cytokines and chemokines. These inflammatory mediators, in turn, boost ROS production, creating a vicious cycle of oxidative stress and inflammation. Prolonged or excessive inflammatory responses can lead to apoptosis of neurons and oligodendrocytes, scar formation, and ultimately, decreased neuronal function [11]. In addition, disruption of the blood–spinal cord barrier (BSCB) allows blood-derived cells and molecules to penetrate spinal cord tissue, causing further damage and worsening oxidative stress [12]. Iron accumulation in injured spinal cord tissue can also contribute to ROS production and oxidative stress. ROS and RNS damage cellular components such as lipids, proteins, and DNA, leading to cell death and tissue damage. Excessive ROS can also interfere with ion channels, leading to intracellular calcium accumulation and excitotoxicity. Furthermore, oxidative stress damages the microvascular endothelium, reduces blood flow to the white matter of the spinal cord, and causes ischemic injury [13, 14].

Acrolein, a byproduct of lipid peroxidation, has been linked to oxidative stress, inflammation, and reactive aldehyde accumulation after SCI [15, 16]. ROS and lipid peroxidases degrade polyunsaturated fatty acids, particularly arachidonic acid [17]. After it is formed, acrolein can bind to multiple cellular macromolecules, such as proteins, DNA, and lipids, leading to their damage and dysfunction [18]. Acrolein can cause inflammation and trigger autophagy-dependent apoptosis by attacking the normal functioning of mitochondria. This leads to the release of cytochrome c from damaged mitochondria, eventually leading to apoptosis. Furthermore, acrolein can penetrate cell membranes and impair vital cellular functions like ion transport and membrane integrity [19].

Overall, oxidative stress plays a significant role in the development of secondary injury following SCI. Antioxidants, including vitamins, can help neutralize ROS and mitigate the damage caused by oxidative stress [14]. For instance, when the body is exposed to acrolein, vitamin C can interact with it and form a compound called AscACR (ascorbate–acrolein adduct). AscACR is a stable product that helps prevent acrolein from reacting with other components in the cell, which can cause damage [20]. Such interventions have the potential to improve functional outcomes after SCI.

## 3. Formation of Glial Scar after SCI

Glial scar formation is a crucial process that occurs after SCI. When the spinal cord is injured, glial cells, such as astrocytes, become activated and migrate to the injury site. These activated glial cells secrete various molecules, including proteoglycans and extracellular matrix proteins like collagen. The glial scar primarily consists of astrocytes and their processes, forming a dense network around the injury site. This scar tissue acts as a physical barrier, preventing further damage and limiting the spread of inflammation. While initially providing some support, the glial scar can also hinder axonal regeneration and functional recovery by creating a barrier that inhibits axon regrowth [21, 22].

Collagen, a fibrous protein, is a major component of the extracellular matrix in scar tissue. It plays a significant role in providing structural support to tissues, including the spinal cord. Collagen forms a network of fibers that offer strength and stability to the injured area. After an SCI, the body produces and deposits collagen at the damage site to rebuild and reinforce the damaged tissue. Collagen formation involves several steps. First, specialized cells called fibroblasts migrate to the injured area. These fibroblasts then produce and secrete collagen molecules, which assemble into a three-dimensional matrix. This matrix acts as a scaffold for other cells involved in the healing process, such as nerve cells and blood vessels. The newly formed collagen matrix creates a favorable environment for tissue regeneration and repair. It provides mechanical support to the damaged spinal cord, promotes cell migration, and guides the growth of new blood vessels. Additionally, collagen helps modulate inflammation and scar formation, allowing for the proper organization of cells during the healing process [23, 24, 25].

Vitamins are essential for collagen synthesis. For instance, vitamin C is necessary for the production of collagen fibers and plays a role in promoting wound healing. It acts as a cofactor for enzymes involved in collagen synthesis and also helps protect collagen from damage [26]. Vitamin B6 is involved in the metabolism of amino acids required for collagen synthesis. It aids in converting the amino acid glycine into hydroxyproline, which is necessary for collagen production [27]. In addition, adequate levels of vitamins A and E are crucial for maintaining the integrity and strength of collagen fibers. They regulate the production of collagenases and protect collagen from oxidative damage [28]. A balanced intake of vitamins is essential for collagen formation and tissue healing following SCI.

## 4. Demyelination after SCI

Demyelination refers to the loss or destruction of the protective covering (myelin sheath) around nerve fibers in the central nervous system (CNS), including the spinal cord. Demyelination following SCI is a complex process involving various mechanisms, including direct trauma, ischemia, inflammation, excitotoxicity, and Wallerian degeneration. These mechanisms can contribute to myelin loss, disrupt nerve signal transmission, and further impair motor and sensory functions. They can also lead to the development of secondary complications, such as spasticity, neuropathic pain, and bladder and bowel dysfunction [29].

Oligodendrocytes are specialized cells responsible for providing myelin sheaths that act as insulation for nerve fibers in the spinal cord, facilitating the efficient conduction of nerve impulses along axons. However, when SCI occurs, oligodendrocytes can be damaged or destroyed, resulting in demyelination. Oligodendrocytes and Schwann cells are responsible for the myelination of axons in the CNS and peripheral nervous system (PNS), respectively. When SCI occurs, oligodendrocytes can be damaged or destroyed, leading to demyelination. Schwann cells can migrate from the PNS to the affected areas of the CNS. These Schwann cells have the ability to myelinate damaged axons in an attempt to restore proper conduction of nerve impulses [30]. However, while oligodendrocytes and Schwann cells both have the potential to contribute to the repair process, there are several factors can hinder their effectiveness. The inflammatory response to injury can release substances, such as tumor necrosis factor *α* (TNF*α*), which inhibits the differentiation and maturation of oligodendrocyte precursor cells (OPCs), responsible for generating new myelin. Proinflammatory cytokines like interleukin (IL)-1*β* and IL-6 can also impair myelin regeneration by interfering with OPC function and survival. Furthermore, immune cells called microglia and macrophages activated during the inflammatory response can release factors that hinder myelin repair, as they can produce ROS and nitric oxide (NO), which are toxic to OPCs and oligodendrocytes. In addition, the formation of scar tissue can create physical barriers that prevent Schwann cells from migrating to the damaged area [31, 32]. Studies have shown that vitamin D deficiency is common in individuals with SCI and has been associated with increased demyelination. Vitamin D Supplementation may help reduce demyelination and promote repair following SCI [33]. Vitamin B12 deficiency is frequently linked to the manifestation of demyelination symptoms, often occurring in the spinal cord. Research has demonstrated that inadequate intake of vitamin B12 during pregnancy can lead to significant impairment in the process of myelination within the nervous system [34]. Hence, it appears that vitamins can have a significant effect on nerve health and potentially contribute to myelin regeneration and repair processes, either directly or indirectly.

## 5. Vitamin A and SCI

All-trans retinoic acid (RA), also known as tretinoin, is an active metabolite resulting from the oxidative metabolism of vitamin A, belonging to the retinoid family. RA plays a vital role in neural differentiation processes in various types of stem cells, enhancing their effectiveness when combined with growth factors [35]. Numerous studies have investigated the application of RA in SCI models. For instance, the combination of mesenchymal stem cell (MSC) transplantation with RA pretreatment has demonstrated improved structural and functional outcomes compared to MSC transplantation alone. RA pretreatment increases the secretion of neurotrophin-3 (NT-3) in MSCs [36]. NT-3 is an essential protein required for the development and survival of neurons in the nervous system. It belongs to a family of proteins known as neurotrophins, which also includes nerve growth factor (NGF), brain-derived neurotrophic factor (BDNF), and NT-4/NT-5. Research has shown that NT-3 plays a significant role in improving neuronal survival and growth, facilitating the regrowth of axons, and sprouting of the corticospinal tract. Moreover, NT-3 promotes the creation of new connections (synapses) between neurons and regulates inflammation while minimizing the formation of scar tissue, which can hinder nerve regeneration [37, 38, 39]. Research by Zhang et al. [40] suggested that a combination of acupuncture and pretreatment with NT3 and RA can enhance the viability and differentiation of transplanted MSCs in rat models of SCI through NT3/tropomyosin-related kinase receptor C (TrkC) signaling. Moreover, RA-primed MSCs have been found to enhance motor activity and reduce tissue damage in post-SCI conditions by inhibiting the high mobility group box 1 (HMGB1)/NF-kB/NOD-like receptor protein 3 (NLRP3) pathway and activating autophagy [41]. In a contusion model of SCI, cotransplantation of olfactory ensheathing cells (OEC) and embryonic stem cell-derived motor neurons (ESMN) treated with RA significantly improved hind limb function in rats [42]. Schrage et al. [43] suggested that RA acts as a signaling molecule for the physiological responses of microglia and neurons after CNS injury. Furthermore, Gao et al. [44] highlighted that the application of nanoparticles made of curcumin and RA has shown potential as nerve regeneration promoters and ROS scavengers for SCI treatment.

One of the consequences of SCI is the disruption of the BSCB, leading to the infiltration of blood cells, an inflammatory response, cell death, and the initiation and exacerbation of secondary damage. Zhou et al. [45] reported that RA significantly reduces BSCB disruption through the activation of autophagic flux and inhibition of cell apoptosis caused by ER stress due to its neuroprotective properties. Another study suggested that systemic administration of RA prevents the SCI-induced increase in proinflammatory cytokine mRNA levels (IL-1*β* and TNF*α*) at 6 hr post-SCI [46]. According to a clinical study, providing SCI patients with a diet rich in vitamin A, carotenoids, zinc, and omega-3 fatty acids over an extended period can significantly reduce inflammation in these individuals [47] (Table 1).

## 6. Vitamin B and SCI

B vitamins are essential coenzymes involved in various enzymatic processes and cellular functions, including those of the nervous system. Each of the B vitamins has positive effects on the central and peripheral nervous systems and is often used in the treatment of various neurological diseases [90, 91].

Anemia is a common issue for the majority of patients diagnosed with SCI, resulting from factors such as blood loss, inadequate nutritional intake, and reduced levels of physical activity. Anemia can lead to a decreased oxygen supply to body tissues, causing symptoms like fatigue, weakness, and shortness of breath. These symptoms can hinder patients' participation in rehabilitation activities and delay their recovery. Therefore, monitoring and managing anemia in patients with SCI are essential for optimizing rehabilitation and improving their overall health [92]. Folic acid (also known as folate, pteroylglutamic acid, or vitamin B9) is an essential water-soluble B vitamin that significantly enhances neuronal function and contributes to maintaining microenvironmental homeostasis in mice with SCI neurons, particularly when combined with mature neural stem cells [51]. Preclinical studies, particularly in rodents, have demonstrated that folic acid, a critical methyl donor in the CNS, enhances axonal regeneration and aids in the repair of damaged CNS, partly through methylation [93]. Matrix metalloproteinases (MMPs), such as MMP2 and MMP9, play an important role in the degradation of the extracellular matrix and the induction of neuropathic pain following SCI [94]. Administration of 80 *µ*g/kg of folic acid has been shown to reduce neuropathic pain and improve motor function after SCI, possibly by downregulating MMP2 [52] and MMP9 [53] expression. Stewart et al. [57] reported beneficial effects of the same dose of folic acid on improving function after a contusion model of SCI. Various studies indicate that folic acid supplementation effectively reduces the incidence of neural tube defects and other congenital anomalies in humans. Iskandar and colleagues, using lesion models of CNS damage, found that the effects of folic acid supplementation on CNS developmental processes are not limited to the embryonic period but can also enhance growth, repair, and recovery in the damaged adult CNS. Their results showed that intraperitoneal treatment of adult mice with folic acid significantly improves the regrowth of spinal cord sensory axons and retinal ganglion cell (RGC) axons to the peripheral nerve graft in vivo. Furthermore, folic acid supplementation enhances neurological recovery from SCI, suggesting its potential clinical impact [95]. After SCI, the neurotoxin acrolein levels increase, leading to neurological deficits. Hydralazine, a drug used to lower blood pressure, has been found to effectively inhibit the negative effects of acrolein after SCI. However, the hypotensive activity of hydralazine can be harmful to neurotrauma patients. To reduce these side effects, Herr [55] and colleagues utilized folic acid-conjugated hydralazine to remove acrolein without significant effects on blood pressure. Polyethylene glycol amine-modified zeolitic imidazole framework-8 nanoparticles containing folic acid (FA-PEG/ZIF-8) have shown potential in aiding the recovery from early-stage SCI by inhibiting proinflammatory microglia/macrophages [56].

In recent decades, B vitamins have been of interest as clinical supplements for treating painful conditions such as neuralgia, diabetic polyneuropathy, and inflammatory diseases. The potential efficacy of B vitamins in managing neuropathic pain carries significant clinical implications. B vitamins are generally considered nontoxic and carry minimal risks compared to potent analgesics like opioids and anti-inflammatory agents. Thiamin (B1) can modify sodium channel conductance to reduce hyperexcitability and thermal hyperalgesia after dorsal root ganglion injury. The active form of B6, pyridoxal phosphate, serves as a coenzyme for *γ*-aminobutyric acid (GABA) synthesis, making B6 bioavailability essential for maintaining inhibitory neurotransmission in pain-related pathways [49, 96]. The injection of a vitamin B complex (i.e., B1, B6, and B12) at the time of ischemic injury and up to 2 weeks later significantly reduced thermal hyperalgesia, although it had no effect on mechanical allodynia [49]. Injection of thiamin (400 mg/kg i.p.) within 24 hr after SCI protects against the consequences of SCI on NO-related amino acids and glutathione in the cerebral cortex [50].

Vitamin B12, also known as cobalamin, is a crucial micronutrient that plays a vital role in DNA and fatty acids synthesis, including myelin. Combination therapy, including vitamin B12, is currently used in clinical settings for patients with neurological diseases. Vitamin B12 can enhance axon formation after traumatic brain injury (TBI) by strengthening stable microtubules and reducing neuronal apoptosis [97]. Recent research suggests that vitamin B12 acts as a superoxide scavenger, promoting axon growth in nerve cells [98]. Biochemical B12 deficiency is common, affecting 7%–19% of patients with SCI. Vitamin B12 deficiency following SCI can result in neurocognitive impairment, often accompanied by other neurological abnormalities. Symptoms may include paranoia, irritability, dementia, hallucinations, psychosis, depression, or confusion [99, 100]. Injection of cyanocobalamin for 3 weeks significantly improved the delirium of a patient with SCI [101, 102]. On the other hand, the administration of B12 for spinal myelopathy was associated with improved motor function and the ability to walk without aids [103, 104]. Case report studies have also indicated improvements in sensory and motor function in patients following vitamin B supplementation [105, 106].

Du et al. [107] observed that following SCI, microbial genes responsible for synthesizing vitamin B6, tryptophan, and folic acid were downregulated. These pathways are vital for proper CNS function [107]. The results of Yang et al.'s studies, investigating various degrees of SCI severity, indicated that the levels of four metabolites—pyridoxine (B6), phosphoriculin, uric acid, and guanidoacetic acid—in cerebrospinal fluid (CSF), plasma, and spinal cord tissue are associated with the injury's severity. These metabolites warrant further exploration to better understand the pathophysiological processes of SCI [108]. Premature coronary artery disease is a leading cause of morbidity and mortality among individuals with SCI. Studies suggest that a high plasma homocysteine level independently increases the risk factor of vascular disease. However, it is recommended that daily intake of folic acid, vitamin B12, or vitamin B6 oral supplements can reduce plasma homocysteine levels [109]. After spinal cord ischemia/reperfusion injury, systemic administration of B vitamins (B1, B6, and B12) has been found to offer neuroprotection and analgesic effects. The analgesic effects of vitamin B treatment may result from reducing or preventing the loss of inhibitory GABAergic tone in the dorsal horn of the spinal cord [54].

Niacin (B3) has demonstrated anti-inflammatory and neuroprotective effects in mice with SCI by targeting the hydroxycarboxylic acid receptor 2 (HCA2) on the surface of macrophages, regulating the phenotypes of infiltrated macrophages, and improving the motor performance of the animal [48]. Additionally, sustained-release niacin monotherapy has proven to be highly effective, safe, and generally well-tolerated for treating dyslipidemia in individuals with SCI [110] (Table 1).

## 7. Vitamin C and SCI

Disruption of vitamin C (ascorbic acid and ascorbate) homeostasis has been linked to the irreversible loss of neuronal function following SCI [111]. After SCI, increased extracellular glutamate levels lead to an accumulation of ascorbic acid. This extracellular ascorbic acid serves two critical protective roles in the CNS. Firstly, it acts as an antioxidant by scavenging oxygen radicals commonly produced following CNS damage, potentially functioning as a defense mechanism against ROS. Secondly, the release of ascorbic acid may coincide with the uptake of excitotoxic glutamate [112]. Lemke et al. [113] found that the levels of endogenous vitamin C decreased following SCI, reflecting potential oxidative reactions.

Among the complications associated with chronic SCI is gallbladder stasis, which can be mitigated through diets containing fiber, vitamin C, vegetable protein, nuts, calcium, and physical activity [114]. One of the treatment-resistant consequences of SCI is axonal degeneration. DNA methylation, modulated by ascorbic acid, may enhance neural regeneration and functional improvement after SCI by controlling the expression of regeneration-related genes [60].

Zhang et al. [58] reported that feeding mice with a mixture of nutrients such as vitamin C, lysine, proline, green tea extract, and more can improve motor dysfunction in mice by reducing the expression of MMP-2 and MMP-9 proteins. Cristante [59] and his colleagues found that the combination of vitamin C and E did not improve motor performance in mice, despite its ability to reduce the inflammatory response. However, a meta-analysis study revealed that the use of vitamin C and vitamin E in animal models of SCI significantly enhanced the recovery of motor performance [5] (Table 2). The coadministration of vitamin C (100 mg/kg) and fluoxetine (1 mg/kg) after SCI helps maintain the integrity of the BSCB and prevent the destruction of tight junction proteins, neutrophil, and macrophage infiltration and increased expression of inflammatory factors and chemokines, and apoptotic cell death. This treatment also improves functional recovery [64]. High-dose ascorbic acid (200 mg/kg) administrated after SCI in rats significantly reduced tissue necrosis and improved functional performance [65, 69]. Treatment with vitamin C at a dose of 20 mg/kg for 5 days effectively protected renal tissues from the systemic effects of SCI, including oxidative stress and inflammation [66]. Rats with SCI treated with taurine and ascorbic acid (at a dosage of 100 mg/kg for 45 days) exhibited significant reductions in markers of apoptosis (caspase-3 and p53), inflammation (IL-6, cyclooxygenase2 (COX2), TNF*α*, and inducible nitric oxide synthase (iNOS)), and oxidative stress (ROS) [67]. In the contusion model of SCI, concurrent injection of ascorbic acid and coenzyme Q10 decreased lipid peroxidation and oxidative stress, enhanced the survival of motor neurons in the anterior horn of the spinal cord, and ultimately improved movement [68]. In a study by Katoh et al. [63] mutant rats lacking the ability to synthesize ascorbic acid showed improvement in movement and tissue preservation after being fed vitamin C and E. Vitamin C eliminated water-soluble free radicals, while vitamin E eliminated fat-soluble free radicals [63].

Based on an in vitro study, the inclusion of ascorbic acid in a culture medium allowed for the prolonged self-renewal of neuroepithelial-like stem cells. This approach also helped to preserve their neuronal pluripotency and expansion capabilities over an extended period, with the cells demonstrating the ability to differentiate into functional neurons. This finding holds promise for regenerative medicine applications in SCI and other CNS disorders by reliably producing large quantities of functional cells [116]. The addition of 0.28 mM L-ascorbic acid phosphate to bone marrow stromal cell culture medium effectively promoted axonal regeneration and improved function by suppressing glial scar formation after SCI, enabling autologous transplantation without the need for scaffolds [61]. In line with cell therapy studies, Salem et al. [62] also suggested that the combined administration of 100 mg/kg vitamin C and bone marrow mesenchymal stem cells (BMMSCs) increased the neuroprotective effects of BMMSCs and reduced the expression of TGF-*β*, TNF*α*, and MMP2 genes. Hamidabadi et al. extracted motor neuron-like cells from hOE-MSCs by adding compounds such as basic fibroblast growth factor (bFGF), fibroblast growth factor 8b (FGF8b), sonic hedgehog signaling (SHH), and ascorbic acid to the culture medium. Transplantation of these cells into SCI rats significantly improved the sensory motor performance of the animals [117].

Individuals with SCI often have difficulty preparing adequately for elective colonoscopy due to prolonged bowel emptying and reduced colonic motility. Korsten et al. [118] showed that a combination of ascorbic acid, polyethylene glycol-electrolyte lavage, and neostigmine can be an effective, well-tolerated, and safe approach to bowel preparation for elective colonoscopy in SCI patients. A 53-year-old male patient with incomplete tetraparesis due to SCI experienced significant improvements in the first 90 days after injury, following treatment with intraspinal erythropoietin (EPO) and subcutaneous administration of granulocyte colony-stimulating factor and vitamin C [119] (Tables 1 and 3).

## 8. Vitamin E and SCI

Vitamin E is a fat-soluble vitamin with eight different forms, with alpha-tocopherol being the most biologically active. It serves as a potent antioxidant in the body, protecting cells from oxidative damage caused by free radicals. Additionally, vitamin E plays a crucial role in maintaining the integrity of cell membranes and regulating gene expression [127].

SCI can have a significant impact on male reproductive health, leading to issues like reduced semen quality, poor sperm function and morphology, and impaired parathyroid function. SCI appears to elevate the levels of ROS in the reproductive system, resulting in oxidative damage to sperm and other reproductive tissues. Vitamins, including vitamin E, serve as powerful antioxidants capable of neutralizing ROS and preventing oxidative damage. Consequently, these vitamins can have a positive effect on male fertility [73]. Studies involving rats have shown that supplementing with vitamin E after SCI can improve semen quality and restore some functions of male accessory glands, such as the prostate and seminal vesicles. These findings suggest that vitamin E holds promise as a therapeutic option to enhance male reproductive health in individuals with SCI [73].

Cervical SCI often results in impaired function of the diaphragm muscle, and individuals with this type of injury may require mechanical ventilation to maintain proper pulmonary gas exchange. Prolonged reliance on mechanical ventilation can, unfortunately, lead to atrophy and dysfunction of the diaphragm. A study conducted by Smuder et al. showed that administering Trolox, a vitamin E analog, before acute SCI can help preserve the contractile function of the diaphragm. This was achieved by reducing proteolytic activation, fiber atrophy, and contractile dysfunction in the diaphragm of experimental animals [74].

Following SCI, various secondary neurochemical processes come into play. These processes involve increased levels of glutamate and intracellular calcium, the degradation of membrane phospholipids, and the production of free fatty acids, often accompanied by the activation of phospholipases and lipases. These cascading events can lead to apoptotic cell death and necrosis, contributing to adverse outcomes observed after SCI [128]. One of the early consequences of these changes in membrane lipid metabolism is a significant reduction in fatty acids, including arachidonate, which acts as a substrate for cyclooxygenase and lipoxygenase. The bioactive products generated from these fatty acids can exacerbate issues such as ischemia, edema, inflammation, and necrosis, ultimately leading to paralysis in SCI cases. In addition, the loss of cholesterol, coupled with an increase in free fatty acids and diglyceride levels, can disrupt cell integrity by altering membrane structure, permeability, and function. Therefore, regulating membrane lipid metabolism becomes a critical aspect of facilitating neuronal recovery following SCI [78, 129]. Studies by Saunders [78] and colleagues demonstrated that pretreatment with a combination of tocopherol (an isoform of vitamin E) and selenium for 5 days before SCI had a significant protective effect on the injured spinal cord tissue by mitigating membrane lipid changes. Subsequent research has supported the antioxidant and/or antilipolytic properties of alpha-tocopherol, which helps preserve spinal cord tissue and improve motor activity [79, 80, 81, 82, 86, 87, 89]. In rat model of SCI, the administration of vitamin E (30 mg/kg four times a day) resulted in a significant decrease in malondialdehyde (MDA) levels, a marker of oxidative stress [76]. Another study reported the positive effects of a dietary supplement rich in alpha-tocopherol (an isoform of vitamin E) over a 2-month period. This supplementation improved motor outcomes, accelerated bladder recovery during urinary retention, and reduced hyperreflexia during the acute phase of SCI. These improvements were associated with the preservation of oligodendrocyte survival and increased supraspinal serotonin levels [77]. Oxidation resistance 1 (OXR1) is an essential factor that helps in maintaining the balance of cells when responding to oxidative stress. In a study conducted by Zhang et al. [75], vitamin E-enriched cationic nanoparticles were used to deliver pOXR1 to rats with SCI (spinal cord injury) models. This innovative therapeutic approach resulted in functional improvement by reducing neuronal apoptosis, decreasing oxidative stress through the Nrf-2/HO-1 pathway, and inhibiting inflammation. Encapsulating alpha-tocopherol in polymeric nanoparticles, as demonstrated in an in vitro study, enhances its antioxidant activity due to improved stability and bioavailability. This finding suggests a promising avenue for SCI treatment [130]. Daily treatment with a dose of 100 mg/kg of vitamin E for 28 days significantly improved hind limb motor function, reduced histopathological changes, and minimized spinal cord tissue damage. This treatment also led to a reduction in oxidative damage markers such as MDA levels and increased the activity of glutathione peroxidase (GPx) [83]. When vitamin E was administered in combination with methylprednisolone after acute SCI, it correlated with a decrease in the extent of ischemic tissue damage and a modest reduction in the concentrations of noradrenaline, adrenaline, and dopamine [84]. Furthermore, after acute SCI, supplementation with vitamin E and trace elements like selenium, zinc, and copper has been shown to help maintain both the number and function of T lymphocytes, which contributes to promoting motor recovery, likely due to its antioxidant effect [85]. Tocotrienol, an isomer of vitamin E, has demonstrated its ability to suppress oxidative stress, inflammation, and iNOS after SCI. These beneficial effects were mediated through the transforming growth factor (TGF)-*β*, type IV collagen, and fibronectin signaling pathways [88] (Table 1).

## 9. Vitamin D and SCI

Individuals with SCI are particularly vulnerable to vitamin D deficiency for various reasons, including insufficient dietary intake, the use of anticonvulsant medications, and limited sunlight exposure [131]. A meta-analysis involving 13 studies with 1,962 patients revealed a high prevalence of vitamin D insufficiency (81.6%) and deficiency (52.5%) in the SCI population [115] (Table 2). Vitamin D plays a critical role in maintaining skeletal health, muscle function, and immune system efficiency. There are two primary forms of vitamin D: D2 and D3. Vitamin D3 (ergocalciferol) is produced in the skin upon exposure to sunlight, while vitamin D2 (total calciferol) is commonly found in dietary sources like fatty fish, egg yolks, and fortified foods. Both forms of vitamin D are biologically inactive and must undergo conversion to the active form, 25-dihydroxy vitamin D (25(OH)2D). However, individuals with SCI may experience reduced mobility and limited sunlight exposure, which can lead to vitamin D deficiency. In addition, some anticonvulsant medications used to manage nerve pain in SCI patients can interfere with vitamin D metabolism, further decreasing vitamin D levels. Dietary restrictions often faced by those with SCI, coupled with nutrient deficiencies and changes in intestinal transit, can also contribute to inadequate vitamin D intake. Vitamin D deficiency can have significant health implications for individuals with SCI, including an increased risk of osteoporosis, musculoskeletal disorders, impaired immune system function, diabetes, pressure ulcers, depression, and cardiovascular diseases [131, 132, 133, 134, 135]. Research suggests that vitamin D supplementation may partially improve muscle strength parameters in individuals with SCI [121]. Moreover, vitamin D analogs have been shown to improve bone mineral density in people with chronic SCI at 6 and 24 months [136]. Subjects with chronic SCI who received a daily dose of 2,000 IU of oral vitamin D3 and 1.3 g of calcium for 3 months were able to achieve normal levels of 25(OH)D [120]. Furthermore, consistent dietary intake of vitamin D has been associated with reductions in total cholesterol levels and improvements in glucose homeostasis in individuals with SCI [125].

Vitamin D supplementation is recommended for enhancing wound healing [137]. Studies have demonstrated the potential benefits of vitamin D in various aspects of SCI recovery. For instance, when rabbits were pretreated with an intraperitoneal injection of 0.5 *μ*g/kg calcitriol, it led to reduced inflammation, inhibition of cell death, and improvements in histopathological, ultrastructural, and neurological scores following SCI [70]. The administration of a daily dose of 800 IU (20 *μ*g) of vitamin D3 over 12 months did not result in significant improvements in serum 25(OH)D levels [124]. Research suggests that higher daily doses of 5,000 or 6,000 IU of vitamin D for 12–16 weeks may be both safe and effective in reaching optimal vitamin D levels in athletes with SCI [122, 123]. Combining vitamin D with other treatments has also shown promise. Twice-daily administration of 0.5 mg/kg progesterone and 5 *μ*g/kg oral vitamin D3 for 5 days in the acute phase of SCI was associated with improved motor and sensory scores after 6 months of treatment [126]. Administration of 1 *μ*g/kg of calcitriol during the acute phase of injury was found to have neuroprotective effects by suppressing the activity of autoreactive lymphocytes against myelin essential protein, a crutial protein for the proper functioning of spinal cord nerve cells. These findings suggest that calcitriol may exert its protective effects by modulating the immune response and targeting the adaptive immune system [71, 72]. Furthermore, research conducted by Li et al. [33] underscores the importance of maintaining adequate levels of vitamin D for preserving myelin integrity after SCI. Their studies showed that vitamin D was cable of rescuing oligodendrocytes from apoptotic cell death and enhancing their ability to myelinate dorsal root axons in vitro. This outcome was achieved by downregulating c-Myc and suppressing the proliferation of oligodendrocyte precursor cells [33]. As a result, it is crucial for individuals with SCI to consistently monitor their vitamin D levels and take steps to ensure sufficient vitamin D intake from dietary sources and appropriate supplementation when necessary (Tables 1 and 3).

## 10. Other Nutrients and SCI

In addition to the previously discussed vitamins, various other nutrients hold promise for individuals with SCI and may provide potential advantages (Table 4)

## 11. Omega-3 Fatty Acids and SCI

Omega-3 fatty acids, a type of polyunsaturated fat commonly found in fish, nuts, and seeds, are recognized for their anti-inflammatory and antioxidant properties. Specifically, eicosapentaenoic acid (EPA) and docosahexaenoic acid (DHA), two essential omega-3 fatty acids, have been found to modulate the immune response, reducing inflammation in SCI [147]. These fatty acids also contribute to tissue repair and regeneration by enhancing cell survival and neuroprotective mechanisms [148]. Research indicates that a diet enriched with omega-3 can improve neurological outcomes post-SCI. Specifically, pretreating animals with a diet high omega-3 polyunsaturated fatty acids resulted in lower sensory deficits, improved bladder function, and enhanced locomotion after SCI. This improvement was associated with correcting docosahexaenoic acid (DHA) deficiency in the spinal cord, highlighting the neuroprotective and restorative effects of dietary omega-3 polyunsaturated fatty acids on the neurolipidome post-SCI [138]. Furthermore, omega-3 fatty acids (435 mg of docosahexaenoic acid and 65 mg of eicosapentaenoic acid) can improve cognitive function and motor activity, which are often negatively affected after SCI [139].

## 12. Zinc and SCI

Zinc, an essential mineral found in foods like oysters, red meat, poultry, nuts, cereals, and dairy products, plays a critical role in various bodily functions. Adequate zinc levels are necessary for proper immune function, tissue repair, and the formation of new blood vessels. Moreover, zinc acts as a cofactor for numerous enzymes involved in cellular metabolism and DNA synthesis, processes vital for nerve regeneration and repair [149]. Notably, after an injury, zinc in the peripheral blood migrates to the central nervous system, making its concentration a reliable biomarker for neurological damage [150]. Research by Xu et al. [140] suggests that zinc supplementation may enhance neuronal function by reducing inflammation, mitigating oxidative stress, and regulating autophagy in SCI. Another study highlights zinc's ability to suppress NLRP3 inflammation after SCI by activating the Nrf2/Ho-1 pathway [141]. Additionally, by regulating the expression of BDNF in the nervous system, zinc contributes to the improvement of neuronal pathology, the regeneration of damaged neurons, and the reduction of neuronal apoptosis [151]. Furthermore, it has been observed that administering zinc can boost the production of granulocyte colony-stimulating factor (G-CSF) by microglia/macrophages (M/Ms), ultimately resulting in reduced levels of neuronal apoptosis following SCI [142]. In summary, maintaining appropriate zinc concentrations can enhance SCI outcomes by inhibiting inflammation, reducing oxidative stress, and protecting damaged neurons.

## 13. Magnesium and SCI

Magnesium, the fourth essential cation in the human body, plays a critical role in numerous cellular functions and enzymes, encompassing metabolic cycles, ion channels, and signaling pathways. Various fundamental and clinical studies have consistently reported a decline in magnesium levels following traumatic CNS injuries. This reduction is considered a significant contributing factor to secondary injury and is often linked to unfavorable neurological outcomes [152]. In the CNS, magnesium's importance lies in its role in maintaining calcium balance. Acting as a natural calcium channel blocker, magnesium helps prevent excessive calcium influx into cells subsequent to an SCI. This, in turn, can mitigate secondary damage and enhance overall outcomes [153]. Conversely, magnesium deficiency is associated with an elevation in plasma substance P concentration (SP) [154]. Substance P stimulates immune cells to release proinflammatory cytokines, such as TNF*α*, IL-1*β*, IL-2, and IL-6, consequently leading to heightened inflammation [155]. Administration of magnesium following an SCI has demonstrated several beneficial effects, including protection against axonal injury [143], reduction in membrane damage, preservation of spinal cord blood barrier integrity [144], normalization of lactate [145], and mitigation of apoptosis [146]. Magnesium holds the potential to serve as a valuable neuroprotective agent, particularly when used in conjunction with other therapies targeting different components of the secondary injury cascade.

## 14. Kinetics of the Vitamins

Vitamins can be broadly classified into two categories based on their solubility: lipid-soluble vitamins and water-soluble vitamins. The kinetics of these vitamins including absorption, distribution, metabolism, and excretion, differ depending on their solubility. Water-soluble vitamins, such as vitamin B and vitamin C, play a vital role in the growth, development, and overall functioning of the human body. Unlike fat-soluble vitamins, water-soluble vitamins are absorbed directly into the bloodstream through the intestinal lining without requiring specific transport proteins. Once absorbed, they are distributed throughout the body via the bloodstream and are not significantly bound to carrier proteins. Water-soluble vitamins are able to move freely in the aqueous compartments of cells. They are metabolized in various tissues and have relatively short half-lives in the body. Excess amounts of water-soluble vitamins are primarily excreted through urine. As these vitamins are not stored significantly, regular intake is necessary to maintain the body's requirements. Water-soluble vitamins are sensitive to factors like light, heat, oxygen, and pH. To protect these vitamins from degradation and enhance their delivery into the body, encapsulation techniques are used [156, 157, 158].

Lipid-soluble vitamins like vitamins A, D, E, and K are mainly present in fatty foods and can dissolve in lipids and fat-based solvents. Because they are lipophilic, they can be stored in the body's adipose tissues and liver. These vitamins are absorbed through the intestinal tract along with dietary fats and need bile salts and pancreatic lipase for digestion and absorption. After absorption, they are incorporated into chylomicrons, which are large lipoprotein particles that enter the lymphatic system before entering the bloodstream. Transport proteins, such as retinol-binding protein for vitamin A and vitamin D-binding protein for vitamin D, carry lipid-soluble vitamins in the bloodstream. Certain proteins assist in transporting vitamins to their intended tissues. Lipid-soluble vitamins undergo metabolism in the liver and other tissues. When dietary intake is insufficient, the body is able to draw from excess vitamin stores in the liver and adipose tissues. However, toxicity can result from excessive intake of lipid-soluble vitamins due to their capacity for storage [159]. Lipid nanocarriers, such as self-emulsifying drug delivery systems (SEDDSs), nanoemulsions, and solid lipid nanoparticles (SLNs), can enhance the oral delivery of vitamins, improving solubility, stability, permeability, bioavailability, and targeting efficiency [160].

Combining both fat-soluble and water-soluble vitamins in one's treatment strategies can be advantageous in meeting nutritional needs and improving overall health. For instance, some vitamins can work together to enhance specific body functions, resulting in a synergistic effect. One example is the combination of vitamin E (soluble in lipids) and vitamin C (soluble in water) which both have antioxidant properties. Interestingly, these two vitamins can regenerate one another, resulting in an overall boost in the body's antioxidant capacity [161]. Some investigators suggest that taking vitamin D and vitamin C supplements together may help promote bone mineralization and prevent bone loss [162]. According to a study on an experimental rat model of peripheral nerve injury, the combination of vitamin E and vitamin B12 was found to be beneficial in improving nerve healing outcomes [163].

## 15. Potential Disadvantage of Excessive Vitamins and Other Nutrients Consumption

It is important to note that while vitamins and nutrients have demonstrated potential benefits for individuals with SCI, they should be consumed as part of a balanced diet and under medical supervision, much like other medical therapies. Individual nutritional needs can vary, and excessive intake may have adverse effects.

Similar to other vitamins, vitamin A can lead to both acute and chronic side effects. Acute retinoid poisoning can result in skin abnormalities and excessive hair loss, while chronic retinoid toxicity can impact multiple organ systems. In terms of bone health, excessive vitamin A intake can lead to the development of bone spurs, calcinosis (abnormal calcium deposits), and bone resorption, potentially increasing calcium levels in the body [164]. Given that individuals with SCI are already at a higher risk of developing osteoporosis and experiencing falls and fractures, prolonged excessive dietary vitamin A intake can exacerbate these issues [165]. As for the vitamin B family and zinc, which are crucial for the growth and function of neurons, excessive consumption can result in nerve damage and neuropathy [166, 167]. Excessive vitamin D intake can lead to its accumulation in the liver and adipose tissue, saturating vitamin D-binding receptors and causing an increase in various other vitamin D metabolites [168]. The most common side effect of excessive vitamin D intake is hypercalcemia, as it enhances calcium absorption in the intestine. Symptoms of hypercalcemia often include weakness, fatigue, loss of appetite, and skeletal discomfort [169, 170]. This is particularly concerning for SCI patients, as it can contribute to balance problems, muscle weakness, osteoporosis, and bone fractures [171, 172]. High doses of vitamin C and E can result in side effects such as gastrointestinal disturbances, including diarrhea, nausea, abdominal cramps, and an increased risk of kidney stones and bleeding. Given the limited mobility of individuals with SCI, these gastrointestinal and kidney issues are more likely to occur [173, 174].

Excessive intake of certain minerals, such as iron, can have negative side effects and potentially worsen inflammation in the body. When the body absorbs and stores too much iron, it can lead to iron overload, also known as hemochromatosis. One of the main concerns with iron overload is its potential to promote oxidative stress. Iron is involved in several biochemical reactions that generate reactive oxygen species (ROS). When there is an excess of iron, these ROS can overwhelm the body's antioxidant defense system, leading to oxidative damage to cells and tissues. Oxidative stress is closely associated with inflammation and is implicated in the development of various chronic diseases [175]. Iron overload has been associated with higher levels of proinflammatory cytokines, which are signaling molecules that participate in immune responses and inflammation. These cytokines can trigger the activation of inflammatory pathways, leading to persistent inflammation. Additionally, iron overload can stimulate the production of inflammatory molecules like C-reactive protein (CRP), which worsens the inflammatory response [176, 177]. According to Feng et al., iron overload in the motor cortex can induce ferroptosis in neurons following SCI. The excess iron in cells increases ROS production, triggering lipid peroxidation and subsequent cell death [178]. Consuming high amounts of long-chain omega-3 fatty acids is associated with an increased risk of developing type 2 diabetes [179], and there is a strong association between SCI and type 2 diabetes [180].

When determining the appropriate vitamin intake for an individual, it is crucial to consider various factors, including age, genetic disorders, medical conditions, and dietary preferences. Each person's nutritional requirements are unique and depend on their specific circumstances. Medical conditions can affect how the body absorbs or metabolizes vitamins, and age can impact the body's ability to utilize nutrients effectively. Additionally, dietary preferences and restrictions play a significant role in determining the right balance of vitamins in one's diet.

## 16. Conclusion

Vitamins play a crucial role in supporting various body functions and maintaining overall health. In individuals with SCI, specific vitamins such as C, E, and A, known for their antioxidant properties, can help reduce inflammation and oxidative stress. These vitamins also contribute to wound healing and tissue repair. B vitamins are essential for converting food into energy and ensuring the proper function of the nervous system. Maintaining adequate levels of vitamin D and calcium is crucial for preserving bone health and strength.

Furthermore, vitamins can have a positive impact on mental well-being and mood. Deficiencies in vitamins like B12 and D have been associated with mood disorders and depression. While vitamins alone may not cure SCI, they play a significant role in supporting bodily functions, optimizing energy levels, and promoting overall health during the recovery process.

It is essential for individuals with SCI to maintain a well-balanced diet. This means consuming a variety of nutrients and vitamins tailored to meet their specific nutritional requirements and support their physical and mental well-being.

## Figures and Tables

**Table 1:** The preclinical studies of vitamins and SCI.

Treatment	Subject	Study design	Effects	Reference
RA + BSA + Cur	RAW264.7 cells, PC12 cells, primary neural stem cell, Mice	T9–T10, 150 *μ*L, 2 mg mL, Tail vein injection, 3 days Transection model	↑ Locomotor function ↓ IL-6, TNF-*α*, and IL-4, CD86, CD206, p-P65, GFAP, *β*3-tubulin, Iba1, CSPGs, M1 ↑ I*κ*B*α*, synapsis remodeling, NeuN, M2	[44]

RA + MSCs	Female SD rats	T9–T10, Transection model	↑ Locomotor function, neuronal differentiation of MSCs NT-3 secretion, axonal regeneration and more neuronal survival	[36]

RA	Female SD rats	T9, 15 mg kg, i.p., 2 weeks Compression model	↑ *β*-catenin, P120, occludin, claudin5, LC3-II ↓CHOP, caspase-12, P62	[45]

RA	Female SD rats	T8, 15 mg kg, i.p. Contusion model	↓ IL-1*β*, TNF*α*	[46]

RA + MSCs + NT-3	Female SD rats	T10, MSCs treated with 10^−6^ mmol/RA and 20 ng ml NT-3 Transection model	↑ Viability and differentiation of transplanted MSCs	[35]

RA + MSCs	Male SD rats	T10, Contusion model	↑ Locomotor function ↓ Beclin-1 and LC3-II HMGB1, p-NF-*κ*B, NLRP3, IL-1*β*, TNF-*α*	[41]

RA + ES	Female Wistar rats	T10–T11 9 days after SCI Balloon compression model	↑ Locomotor function, neural regeneration, ESMN survival	[42]

Vitamin B3	C57/BL6 mice M1 macrophages	In vivo: T8–T10, 100 mg kg, p.o., contusion model In vitro: 300 *μ*M	↑ MBP, CD206, IL-10, IL-13, locomotor function ↓ p65 NF-*κ*B, CD86, IL-12, IL-6, demyelination	[48]

Vitamin B	—	L4–L6, 2 weeks, Ischemic model	↓ GFAP, Iba1	[49]

Vitamin B1	Female SD rats	T8–T10, 400 mg kg, i.p., 24 hr after SCI Contusion model	↑ Lys/Gly ratio, GSH ↓ Cit/Arg, Cit/Orn ratio	[50]

NSC transplantation + vitamin B	Male SD rats	T9–T11, Contusion model	↑ Locomotor function, repair of injured spinal cord tissues, BCL-2, GDNF, BDNF, and NT-3 ↓ Bax, caspase-3	[51]

Vitamin B	Male SD rats	T9, 80 *µ*g/kg, i.p., 3 days prior and 17 days post-SCI (mid-phase) 3 days pre-cSCI and 14 days post-SCI ending on the 42nd day of SCI (late phase)	↑ Locomotor function ↓ MMP2, MMP9, neuropathic pain	[52, 53]

Vitamin B1, B6, and B12	Male SD rats	33, 33, 0.5 mg kg, i.p 7–14 days Ischemia/reperfusion SCI model	↑ GAD, GABA, *β*-III-tubulin ↓ Thermal hyperalgesia, vanilloid receptor 1, c-Fos, GFAP, Iba1	[54]

Hydralazine conjugated to vitamin B	Male SD rats	T10, 5 mg kg, i.v., contusion model	Maintaining normal blood pressure ↓ Acrolein levels	[55]

Vitamin B	Male C57BL/6 mice	T9-T10, i.v., Contusion model	↑ Locomotor function, IL-10 ↓ iNOS, Iba1, IL-1*β*, M Ms	[56]

Vitamin B	Male and female SD rats	80 *μ*g/kg, 3 days before and 17 days after SCI Contusion model	↑ Locomotor function	[57]

NM (700 mg vitamin C, L-lysine, L-proline, L-arginine N-acetyl cysteine, standardized green tea extract, selenium, copper, and manganese)	Male CD1 mice	T6-T7, 500 *µ*g and 2,000 *µ*g, p.o., 3 days Compression model	↓ MMP-2, MMP-9 ↑ Locomotor function	[58]
Vitamin C + vitamin E	Male Wistar rats	T9-T10, 100 mg/kg, 4 weeks Contusion model	Did not improve their neurological performance ↓ Inflammatory response	[59]

Vitamin C	Female SD rats	T9, 5, 100, or 200 mg kg, i.p., 12 weeks Contusion model	↑ Locomotor function, axonal sprouting within the lesion cavity of the spinal cord ↑ 5hmC, Tet1, Tet2, Tet3	[60]

Vitamin C	Female Fischer 344 rats	T8, 0.28 mM Transection model	↑ Locomotor function, Tuj1, axonal regeneration ↓ GFAP	[61]

BMMSCs + vitamin C	Albino male rats	T9-T10, 100 mg kg Contusion model	↑ NF200, nestin, locomotor function ↓ TGF-*β*, TNF*α*, MMP2	[62]

Vitamin C + vitamin E	Shionogi rats	T12 91 IU kg, 55 mg kg Compression model	↑ Locomotor function	[63].

Vitamin C + fluoxetine	Male SD rats	T9, 100 mg kg, 2 weeks Contusion model	↑ Locomotor function, ZO1, occludin ↓ Disruption, caspase 3, MMP9, Cox2, iNOS, IL-1*β*, TNF-*α*, ED1, Gro-*α*, MIP-1 *α*, MIP-1*β*	[64]

Vitamin C	Female SD rats	T8–T12, 100, 200 mg (kg) i.p., 4 weeks Contusion model	↑ Locomotor function ↓ Tissue necrosis	[65]

Vitamin C	Male Wistar rats	20 mg kg, 5 days Contusion model	↑ GSH, SOD, CAT, GST, GPx ↓ ROS, lipid peroxidation, protein carbonylation, protein sulphydryl, NF-*κ*B, COX- 2, iNOS, MPO, IL-1*β*, TNF-*α*	[66]

Vitamin C + taurine	Male albino Wistar rats	T9-T11, 100 mg kg, 45 days Contusion model	↑ GSH, SOD, CAT, GPx ↓ Caspase-3, p53, COX-2, iNOS, MPO, IL6, TNF-*α*, ROS, MDA	[67]

Vitamin C + coenzyme Q10	Male Wistar rats	T8-T9, 100 mg kg, 10 mg kg, i.p., 24 hr after SCI Contusion model	↑ Locomotor function, motor neurons survival, GPx ↓ MDA	[68]

Vitamin C	SD rat	T11, 200 mg kg, i.p., 30 min after SCI	↑ Locomotor function ↓ Necrosis and bleeding	[69]

Calcitriol	Rabbits	0.5 *μ*g/kg, i.p.,7 days before SCI Ischemia reperfusion SCI model	↑ CAT, normal motor neurons ↓ Caspase-3, MPO, XO, MDA	[70]

Calcitriol	Female SD rats	1 *μ*g/kg, 7 days	↑ Locomotor function ↓IFN-*γ*, IL-4, IL-17 A, leukocyte infiltration, moto neuronal loss	[71]

Vitamin D3	SD rats	T7–T9, 1 *μ*g/kg, i.p., On days 1, 3, 5, 7	↑ Locomotor function	[72]

Vitamin D3 + calcium+ phosphorus + picolab	Male SD rats Male C57/6N mice	T9–T11, 2.3 IU/g, 3 weeks Contusion and transection model	↑ Locomotor function, Preserved myelin sheath integrity ↓ c-Myc, apoptosis	[33]

Vitamin E	Male SD rat	2, 10 mg kg, p.o., 8 weeks Contusion and Transection model	↑ Sperm motility, sperm viability, mitochondrial potential, sperm cAMP content, sperm protein phosphorylation, prostate weights, seminal vesicle weights ↓ Sperm head swelling	[73]

Trolox	Female SD rats	C2, 20 mg kg, i.p., immediately after SCI and every 12 hr until euthanasia Hemisection model	↓ Atrogin-1/MaFbx, MuRF1, LC3, ERK	[74]
Cationic nanoparticles (vitamin E succinate-grafted *ε*-polylysine) + OXR1 plasmid + liposomes	Female SD rats	T9–T10 Contusion model	↑ Locomotor function, OXR1, Bcl-2, Nrf2, HO-1, CAT, SOD ↓ Bax, cleaved caspase3, laminin, neurocan, CD68	[75]

Vitamin E	Male Swiss albino rats	T3, 30 mg kg, 4 times/day Compression model	↓ MDA	[76]

Vitamin E	Female SD rats	T8-T12, 51 IU/g, 8 weeks Contusion model	↑ Locomotor function, bladder function, preserve neurons and oligodendrocytes, serotonin ↓ Depression	[77]

Vitamin E	Mongrel cats	L1-L3, 1,000 IU, p.o., 5 days before SCI Compression model	↓ 25-Hydroxycholesterol, PGE2, thromboxane B2, prostacyclin	[78]

Vitamin E	Mongrel cats	L2, 1,000 IU, p.o., 5 days before and 5 after SCI Compression model	↑ Locomotor function	[79]

Vitamin E	Male Wistar rats	T12, 2 and 50 IU/100 g, 8–10 weeks Compression model	↑ Locomotor function, maintaining normal blood flow and arachidonic acid metabolism ↓ MDA	[80, 81, 82]

Vitamin E	Male SD rats	T11-L1, 100 mg kg, p.o., 4 weeks Transverse apophyses model	↑ Locomotor function ↓ MDA, GPx, cavity volume size	[83]

Vitamin E + MP	SD rats	C8–T1, 100 U kg Compression model	↓ Noradrenalin, adrenaline, dopamine, ischemic area	[84]

Vitamin E + zinc + copper + selenium	Female SD rats	T9, 50 mg kg, 15 days Contusion model	↑ Locomotor function, T-cell count, T-cell stimulation index	[85]

Vitamin E	Female SD rats	T7-T8, 1,000 and 2,000 mg kg, p.o., 2 weeks Compression model	↑ Locomotor function	[86, 87]

Vitamin E	Female SD rats	8 weeks Compression model	↑ Locomotor function, SOD, CAT, GSH ↓ MDA, NF-*κ*B, TNF-*α*, IL-1*β*, IL-6, iNOS, TGF-*β*, collagen type IV, fibronectin, cavity volume size	[88]

Vitamin E	Female Wistar rats	C3-C9, 100 mg kg, 1 week Compression model	↓ MDA	[89]

Arg, L-arginine; Bax: Bcl-2-associated X protein; Bcl-2, B-cell lymphoma 2; BDNF, brain-derived neurotrophic factor; BSCB, blood–spinal cord barrier; C, cervical; CAT, catalase; CHOP, C/EBP homologous protein; Cit, L-citrulline; COX-2, cyclooxygenase-2; CSPGs, chondroitin sulfate proteoglycans; ERK, extracellular signal-regulated kinase; ESMN, embryonic stem (ES) cell-derived motor neurons; GABA: gamma-aminobutyric acid; GAD, glutamic acid decarboxylase; GDNF, glial cell line-derived neurotrophic factor; GFAP, glial fibrillary acidic protein; Gly, glycine; GSH, glutathione; GST, glutathione-S-transferase; GPx, glutathione peroxidase; Gro, growth-regulated oncogene; 5hmC, 5-hydroxymethylcytosine; HMGB, high mobility group box; HO-1, heme oxygenase 1; Iba1, ionized calcium binding adaptor molecule 1; IL, interleukin; IFN-*γ*, interferon-gamma; iNOS, inducible nitric oxide synthase; i.m., intramuscular; i.v., intravenous; L, lumbar; LC3, light chain 3; MSCs, mesenchymal stem cells; MDA, malondialdehyde; MIP, macrophage inflammatory protein; MMP, matrix metalloproteinases; MP, methylprednisolone; MuRF1, muscle RING finger 1; MPO, myeloperoxidase; NF-*κ*B, nuclear factor kappa B; NT-3, neurotrophin-3; NF, neurofilament protein; Nrf2, nuclear factor erythroid 2-related factor 2; 25(OH)D, 25-hydroxy vitamin D; Orn: L-ornithine; OXR1, oxidation resistance 1; PGE2, prostaglandin E2; p.o., oral administration; RA, retinoic acid; ROS, reactive oxygen species; SCI, spinal cord injury; SD, Sprague–Dawley; SOD, superoxide dismutase; T, thoracic; TGF-*β*, transforming growth factor *β*; TNF-*α*, tumor necrosis factor.

**Table 2:** The systematic review studies of vitamins and SCI.

Aim	Search databases	Types of studies included	No. of studies included	Review dates	Results	Reference
To investigate the effects of vitamin C and vitamin E on recovery of motor function after spinal cord injury in animal models	EDLINE, Embase, Scopus, Web of Science	Animal models studies	10	Through November 2018	(1) Vitamin C and vitamin E improved motor function recovery in animals with spinal cord injury (2) Vitamin C is effective when given intraperitoneally (3) Concurrent supplementation with both vitamins did not show better efficacy than using either one alone	[5]

To investigate the impact of vitamin D on the prognosis after spinal cord injury	Medline, Embase, Scopus, Web of Science	Clinical studies	35	1974 to August 31, 2021	(1) Vitamin D deficiency is prevalent in the SCI population (2) Low vitamin D levels are associated with complications that hinder functional restoration after SCI (3) Vitamin D supplementation may accelerate rehabilitation after SCI, but further research is needed	[115]

**Table 3:** The clinical studies of vitamins and SCI.

Treatment	Subject	Study design	Effects	Reference
Vitamin B3	Men and women 18–65 years	Complete SCI and C4–C8 levels for longer than 1 year. Forty-eight-week Dose of extended release niacin started at 500 mg nightly before bedtime for 4 weeks and then increased in 500-mg increments every 4 weeks to a peak dose of 2 g before bedtime	↑ HDL-C ↓ TC/HDL-C ratio, LDL-C/HDL-C ratio, LDL-C, TC, TG	[110]

Vitamin B	Man, 48-year-old	C6, 16 weeks	↑ Locomotor function	[105]

Vitamin B12	Man, 57-year-old	1,000 *µ*g, 5 days, and 1,000 *µ*g, once a week for the following 3 months, 1,000 *µ*g/month, i.m.	Improve intraspinal myelon lesion and hypesthesia of both arms	[106]

Vitamin C	27 chronic SCI 18 to 69 years	—	Improves bowel preparation for elective colonoscopy	[118]

Vitamin C + G-CSF	53-year-old male	C5–C7, T1, subcutaneous every 3–5 days for 58 days	↑ Locomotor function	[119]

Vitamin D3 + calcium	7 subjects	2,000 IU, p.o., 12 weeks	↑25(OH)D	[120]

Vitamin D3	5 subjects	25,000 IU, 2 weeks/8 weeks Double blinded, randomized pilot trial	Improve muscle strength	[121]

Vitamin D3	10 men SCI athletes, 18–60 years	6,000 IU, 12 weeks	Maintaining optimal 25(OH)D levels	[122]

Vitamin D3	9 men and women SCI athletes, >18 years	5,000 IU, 16 weeks	Maintaining optimal 25(OH)D levels	[123]

Vitamin D3	50 subjects	50 *μ*g twice a week, for 14 days 800 IU (20 *μ*g) daily for 12 months	Little effect on improving serum 25(OH)D levels	[124]

Vitamin D	20 men 18–65 years	5.33 ± 4.14 *µ*g, 3 days	↑ Glucose homeostasis ↓ Total cholesterol	[125]

Vitamin D3 + progesterone	19 men and 14 women 18−65 years	5 *μ*g/kg, p.o., twice/5 days Randomized, double-blind, placebo controlled	↑ Locomotor and sensory function	[126]

C, Cervical; G-CSF, Granulocyte-colony stimulating factor; HDL, High-density lipoprotein; LDL-C, Lipoprotein-cholesterol; 25(OH)D, 25-Hydroxy Vitamin D; p.o., Oral administration; SCI, Spinal cord injury; T, Thoracic; TC, Total cholesterol; TG, Triglycerides.

**Table 4:** The other nutrients and SCI.

Treatment	Subject	Study design	Effects	Reference
Omega-3 polyunsaturated fatty acids	Female SD rats	T10, 750 mg kg, 8 weeks Contusion model	↑ Locomotor function, Bladder function, neurolipidome Restore the DHA status ↑protein kinase B/Akt and CREB	[138]

Omega-3 fatty acid	44 men and 10 women	Docosahexaenoic acid (435 mg) and eicosapentaenoic acid (65 mg), p.o.,14 months	↑ Locomotor function and cognitive function	[139]

Zinc	C57BL/6 mice	T8–T9, 10, 30, 50, 100 mg kg, i.p. Contusion model	↑ Locomotor function ↑Atg5, LC3B, SIRT3, SOD, Nrf2 ↓ IL-1*β*, TNF*α*, IL-6, NLRP3, caspase-1, ROS, MDA	[140]

Zinc	C57BL/6 J mice	30 mg kg, i.p., 2 hr after SCI Contusion model	↑ Locomotor function, neural survival ↑ SOD, GSH, Nrf2, HO-1, NQO-1 ↓ MDA, NLRP3, IL-1*β*, caspase-1	[141]

Zinc	C57BL/6J mice	T9, 30 mg kg, 3 days Contusion model	↑ Locomotor function, neural survival ↑ G-CSF, NF-*κ*B, Bcl-2 ↓ Bax, caspase-3	[142]

Magnesium	Rats	T9, 300 and 600 mg kg, subcutaneous, 1 hr after SCI Compression model	↓ MDA	[143]

Magnesium	Female SD rats	T7–T8,100 and 600 mg kg, i.p. Contusion model	↑ Locomotor function, neuroprotection on spinal cord ultrastructure	[144]

Magnesium	Rabbits	100 mg kg	Normalization of lactate ↓ MDA	[145]

Magnesium	Female Wistar albino rats	T7–T9, 600 mg kg, i.p. Contusion model	↓ Caspase-3	[146]

ATG5, autophagy-related 5; Bax, Bcl-2-associated X protein; Bcl-2, B-cell lymphoma 2; DHA, dicosahexaenoic acid; G-CSF, granulocyte colony-stimulating factor; GSH, glutathione; HO-1, heme oxygenase 1; IL, interleukin; LC3B, light chain 3 B; MDA, malondialdehyde; NF-*κ*B, nuclear factor-kappa B; NLRP3, nucleotide-binding domain, leucine-rich–containing family, pyrin domain–containing-3; NQO1, NAD (P)H quinone oxidoreductase 1; Nrf2, nuclear factor erythroid 2-related factor 2; ROS, reactive oxygen species; SCI, spinal cord injury; SD, Sprague–Dawley; SIRT3, sirtuin 3; SOD, superoxide dismutase; T, tthoracic.

## Data Availability

No underlying data was collected or produced in this study.
